# Gut Bacterial Characteristics of Patients With Type 2 Diabetes Mellitus and the Application Potential

**DOI:** 10.3389/fimmu.2021.722206

**Published:** 2021-08-12

**Authors:** Yanyan Que, Man Cao, Jianquan He, Qiang Zhang, Qiongyun Chen, Changsheng Yan, Aiqiang Lin, Luxi Yang, Zezhen Wu, Dan Zhu, Fengwu Chen, Zhangran Chen, Chuanxing Xiao, Kaijian Hou, Bangzhou Zhang

**Affiliations:** ^1^Department of Endocrinology, Zhongshan Hospital Xiamen University, Xiamen, China; ^2^Department of Mathematics and Numerical Simulation and High-Performance Computing Laboratory, School of Sciences, Nanchang University, Nanchang, China; ^3^Department of Rehabilitation, Zhongshan Hospital Xiamen University, Xiamen, China; ^4^School of Pharmacy, Fujian University of Traditional Chinese Medicine, Fuzhou, China; ^5^Department of Gastroenterology, Zhongshan Hospital Xiamen University, Xiamen, China; ^6^School of Medicine, Xiamen University, Xiamen, China; ^7^Department of Research and Development, Xiamen Treatgut Biotechnology Co., Ltd., Xiamen, China; ^8^Department of Endocrine and Metabolic Diseases, Longhu Hospital, The First Affiliated Hospital of Medical College of Shantou University, Shantou, China; ^9^Graduate School, Medical College of Shantou University, Shantou, China

**Keywords:** microbiota, meta-analysis, T2DM, probiotics, 16S rRNA sequencing

## Abstract

Type 2 diabetes mellitus (T2DM) is a complex disorder comprehensively influenced by genetic and environmental risk, and research increasingly has indicated the role of microbial dysbiosis in T2DM pathogenesis. However, studies comparing the microbiome characteristics between T2DM and healthy controls have reported inconsistent results. To further identify and describe the characteristics of the intestinal flora of T2DM patients, we performed a systematic review and meta-analysis of stool microbial profiles to discern and describe microbial dysbiosis in T2DM and to explore heterogeneity among 7 studies (600 T2DM cases, 543 controls, 1143 samples in total). Using a random effects model and a fixed effects model, we observed significant differences in beta diversity, but not alpha diversity, between individuals with T2DM and controls. We identified various operational taxonomic unit (OTUs) and bacterial genera with significant odds ratios for T2DM. The T2DM signatures derived from a single study by stepwise feature selection could be applied in other studies. By training on multiple studies, we improved the detection accuracy and disease specificity for T2DM. We also discuss the relationship between T2DM-enriched or T2DM-depleted genera and probiotics and provide new ideas for diabetes prevention and improvement.

## Introduction

According to the 2019 Ninth International Diabetes Federation Diabetes Atlas, there are approximately 463 million diabetic patients worldwide (1). It is expected that the number of diabetic patients will increase from 578.4 million in 2030 to 700.2 million in 2045, representing an increasing public health threat throughout the world (1). Epidemiologically, Type 2 diabetes mellitus (T2DM) characterized by glucose intolerance accounts for approximately 90% of all diabetic patients worldwide (2, 3), and is a complex multifactorial metabolic disorder involving genetic (e.g. *Tcf7l2*, *Kcnq1*) and environmental lifestyle factors (e.g. intake of energy-dense refined food, sedentary behavior) (4–7). Meanwhile, the imbalance between immune cells results in the production of excess chemokines and proinflammatory cytokines that promote systemic inflammation and lead to peripheral insulin resistance. Subsequently, this immunological dysfunction leads to diabetic patients being more risky toward many infectious diseases (diabetic foot, diabetic nephropathy, et al.) (8, 9). Therefore, the study of pathological mechanisms is of great significance for the effective prevention and treatment of T2DM.

With the development of high-throughput sequencing technology, increasing evidence has shown that gut microbiota dysbiosis, as an important environmental factor, may lead to diabetes (10–15). Microbial diversity indexes including the phylogenetic diversity and Chao1 were significantly decreased in T2DM (16). Studies have also revealed that the gut microbiome of T2DM is characterized by an enrichment of opportunistic pathogens (11) and sulfate-reducing bacteria (17, 18) and depletion of probiotics (19) and butyrate-producing bacteria (11, 17, 18, 20). For example, butyrate-producing *Roseburia* has been shown to causally improve glucose tolerance (21, 22). Wu et al. found that *Bifidobacterium* and *Bacteroides* were less represented in the diabetic group than in the nondiabetic group (19). A Chinese study suggested that *Clostridium coccoides* and *Clostridium leptum* were significantly lower, while the fecal count of *Lactobacillus* was significantly higher in diabetic patients than in healthy controls (23), which is in line with previous literature indicating that *Lactobacillus* might contribute to chronic inflammation in diabetes development (10, 24). Moreover, several studies have investigated the effects of modulation of gut microbiota on improvements of T2DM. A randomized, double-blind, and placebo-controlled study (25) showed that consumption of yogurt containing *Bifidobacterium lactis* BB-12 and *Lactobacillus acidophilus* LA-5 for 6 weeks significantly reduced the levels of blood glucose and glycated hemoglobin (HbA1) and increased the levels of erythrocyte superoxide dismutase (SOD) and glutathione peroxidase (GPx) activity and total antioxidant capacity. Similarly, the blood glucose, insulin, homeostasis model assessment for insulin resistance (HOMA-IR) index and inflammation were significantly reduced by probiotic intervention in a randomized double-blind placebo-controlled study of 61 Saudi T2DM patients (26). Recently, Mocanu et al. found that fecal microbiota transplantation (FMT) combined with low-fermentable fibers interventions regulated gut microbiota and improved HOMA2-IR and insulin sensitivity of obesity and metabolic syndrome patients (27). Therefore, gut microbiota dysbiosis is associated with T2DM, and gut microbial modulation is likely an effective strategy to improve T2DM by precision supplement of probiotics and even FMT.

Although many studies have monitored the gut microbiota and investigated its relationship with T2DM in different populations (28–32), inconsistent results describing microbial differences have been reported between diabetic and healthy individuals. For example, Larsen et al. found that the proportions of the phylum Firmicutes and class Clostridia were significantly reduced in T2DM patients compared to the control group (10); whereas one Pakistani study with 60 individuals revealed that bacteria from Firmicutes along with those from Clostridia and Negativicutes were predominant in obese T2DM patients (28). On the other hand, Doumatey et al. reported a significantly lower richness in T2DM (30), while Ahmad et al. and Chávez-Carbajal et al. observed no significant difference in the alpha diversity index observe (28, 29). In short, the key issue associated with the gut microbiota differences between T2DM and healthy controls is the lack of apparent reproducibility in different studies when identifying the microbiome characteristics in T2DM.

Here, we systematically reviewed, collected, and analyzed 16S rRNA gene raw sequencing data from 7 studies that investigated the intestinal microbiome of T2DM patients in relation to controls, and performed a meta-analysis on gut bacterial alpha-diversity, beta-diversity, community composition, as well as the analyses of classification model and bacterial correlation. We were aiming to better understand the gut microbe differences between T2DM patients and controls across countries, develop a complementary approach for the risk assessment of T2DM, and reveal the potential of probiotic therapeutic measures for T2DM from the perspective of intestinal microecology.

## Materials and Methods

### Database Search and Study Selection

In adherence with the Preferred Reporting Items for Systematic Reviews and Meta-Analyses (PRISMA) guidelines (33), a systematically computerized literature search of PubMed, EMBASE, and Web of Science was conducted until May 2020. The search strategy was as follows: diabetes (T2DM) and fecal microbiota and human and 16S rRNA. Additionally, the reference lists of identified original articles and reviews were reviewed manually for potential studies that might have been missed during the search. After an overview of the titles and abstracts, 22 publications were retained for further review of the full texts (**Table S1**). Studies were finally included if they met the following inclusion criteria: 1) studies were based on human fecal samples from T2DM patients and healthy subjects; 2) samples were sequenced by NGS for the 16S rRNA gene; and 3) raw sequencing data, barcodes, and metadata were publicly available or provided by the authors until October 20, 2020 upon request by email. Finally, sequencing datasets and metadata from 7 studies were obtained for subsequent analyses (16, 28–32), excluding the other 15 studies due to incomplete information on sequences, barcodes, or metadata (10, 15, 23, 34–44) (**Table 1**). The baseline clinical characteristics of participants recruited in the 7 studies were summarized in **Table S2**. The other five data-sets downloaded for model validation were generated from patients who suffered from the following diseases: colorectal cancer (CRC) (45), Parkinson (46), inflammatory bowel disease (IBD) (47), non-alcoholic fatty liver disease (NAFLD) (48), and fat syndrome (49).

**Table 1:** Characteristics of the data sets included in the fecal sample-based analysis.

Source	Year	Country	HC	T2DM	DNA extraction	Region	Seq platform
PRJNA325931	2016 (32)	Colombia	84	28	QIAamp DNA	V4	Miseq
					Stool Mini Kit		
SRP168691	2019 (31)	China	35	65	FastDNA Spin Kit	V3-V4	Ion S5
PRJNA554535	2019 (28)	Pakista	20	40	Tiagen DNA	V3–V4	Miseq
					Stool kit		
PRJNA472187	2020 (29)	Mexico	76	68	PowerSoil DNA	V3	PGM
					Isolation Kit		
PRJNA607849	2020 (30)	Nigeria	193	98	MoBioPowerMag	V4	Miseq
					Microbiome kit		
ERP107659	2020 (16)	China	40	20	QIAamp DNA	V4-V5	Hiseq
					Stool Mini kit		
PRJNA670300	2020	China	95	281	QIAamp DNA	V4	Miniseq
					Stool Mini kit		

### Microbiome Data Processing

The V4 or V3-V4 region of the 16S rRNA gene was the most frequently sequenced fragment with the Illumina (MiSeq or HiSeq) or Ion Torrent platform (PGM or S5) among the included studies (**Table 1**). Despite the different sequencing platforms and hypervariable regions of the 16S rRNA gene, we applied a uniform analytical pipeline to minimize the impact of these differences. Briefly, raw reads were quality filtered by Usearch (50) with -fastq_maxee 0.5 or were assembled using FLASH (v1.2.11) by with -x 0.2 and -M 200 for V3-V4/-M 250 for V3-V5/-M 150 for the V4 region. Closed-reference OTU picking at 97% identity was performed with Usearch against the SILVA132 database (51). For all taxonomic and diversity analyses, samples with sequencing depths less than 10000 sequences in the OTU table were not used for downstream analyses. The OTU table was rarefied to the lowest sequencing depth within each study.

### Statistical Analysis

The α diversity indexes, bacterial richness (observed OTUs), Shannon index, and evenness (J) were calculated based on OTU tables of each study. Significance tests between T2DM patients and healthy controls were conducted by the Wilcoxon test method. Differences in community structure across samples (β diversity) were visualized by principal coordinates analysis (PCoA) plots based on Bray-Curtis distance. Significance tests were determined using permutational multivariate analysis of variance (PERMANOVA) with 10^4^ permutations in vegan (52). Meta-analysis of bacterial alpha diversity indexes and microbial taxa among the 7 studies was performed to determine the consistency using both the random effects (RE) model and fixed effects (FE) model in the metafor package (53). Generally, we calculated the odd ratios (ORs) of these metrics by assigning any value above the median of the metric within the study as positive.

Random forest (RF; number of trees, 500) models were trained for individual studies, and datasets combined all studies together at the OTU and genus levels to test whether a mixture of featured taxa can predict T2DM. We evaluated their performance using leave-one-out (LOO) cross-validation and scored the predictive power in a receiver operating characteristic (ROC) analysis. Meanwhile, to refine microbiome signatures for diabetic detection, we developed a two-step procedure modeling workflows with rigorous external validation to avoid overfitting and overoptimistic reports of model accuracy. In the first step, we ranked the common OTUs and genera by their relative abundances. Next, as a precaution against overoptimistic evaluation, stepwise feature selection was employed to select predictive microbial features and eliminate uninformative features based on 10-fold cross-validation (the depict in **Figure S1**). The discriminatory power of OTUs and genera was calculated as the area under the ROC curve (AUC). Subsequently, we further explored the interaction between different genera and probiotics by Cytoscape (v3.5.1) (54). All statistical and correlation analyses were conducted in R (v3.5.3) (55). Figures were plotted mainly used ggplot2 (v3.0.0) (56) and gridExtra (57).

## Results

### Characteristics of Included Studies

**Figure 1:**
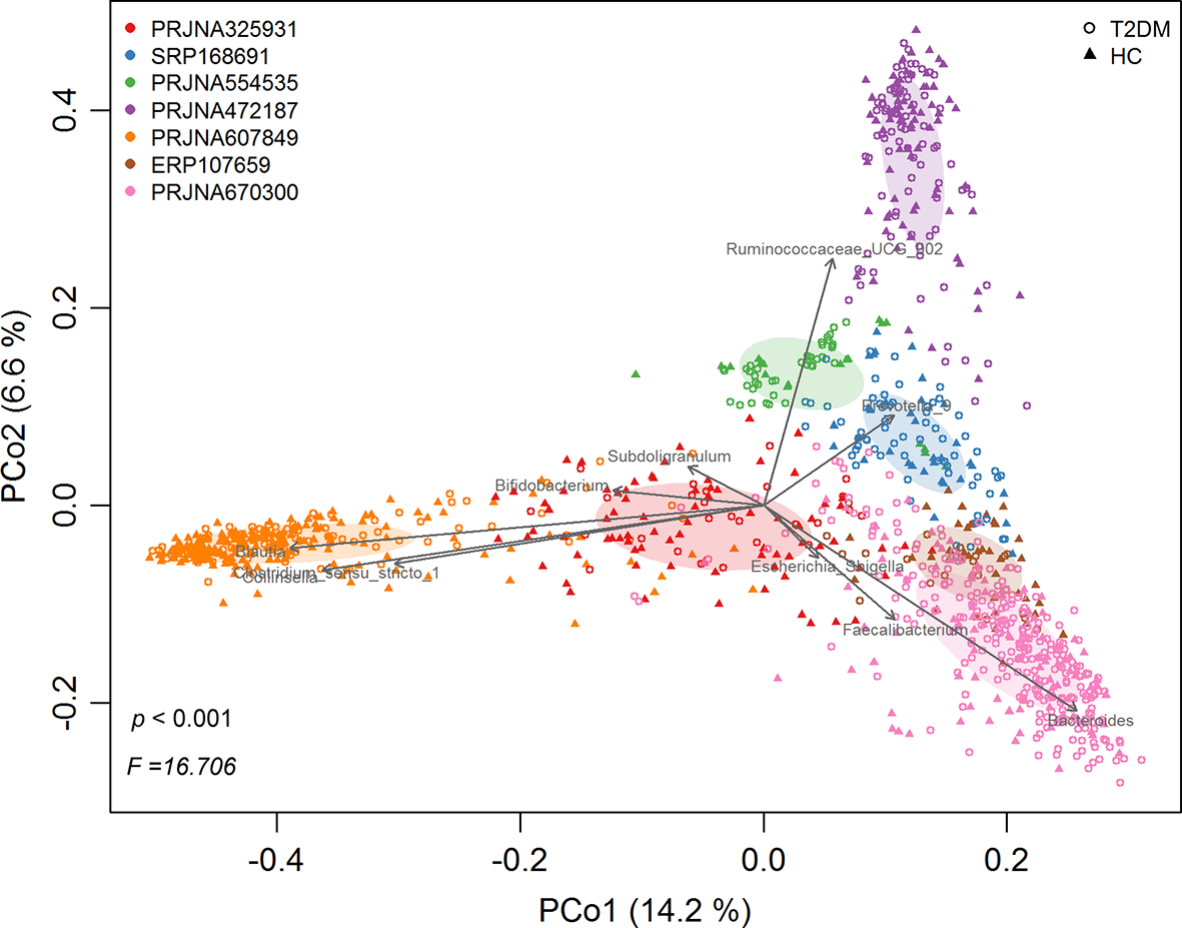
The principal coordinates analysis (PCoA) of all samples at OTU level, depicting the great microbial variations from different studies with population variation, DNA extraction methods, 16S rRNA gene regions investigated, sequencing platforms, etc. The points represent samples, shapes represent the different group, and the colors represent the different study. Top 10 genera with significant (*P < 0.001, p < 0.05*) correlations were fitted to the PCoA.

Following quality filtering, a total of 1143 samples (543 healthy controls and 600 T2DM patients) from 7 studies were retained for downstream analyses (**Table 1**). Overall gut microbial community structures in T2DM patients were significantly different from those in healthy individuals (PERMANOVA, F=16.706, *p*<0.001) when combining all samples from the 7 individual studies together. However, samples were distinctly clustered primarily by individual studies in PCoA (**Figure 1**), probably due to different populations (ethnicity) worldwide, as well as strong variables such as DNA extraction methods, 16S rRNA gene regions investigated, and sequencing platforms adopted by individual studies. This large variability in the gut microbiota across studies prompted us to perform a further meta-analysis.

### Microbiome Profile Differences Between T2DM and Controls

**Figure 2:**
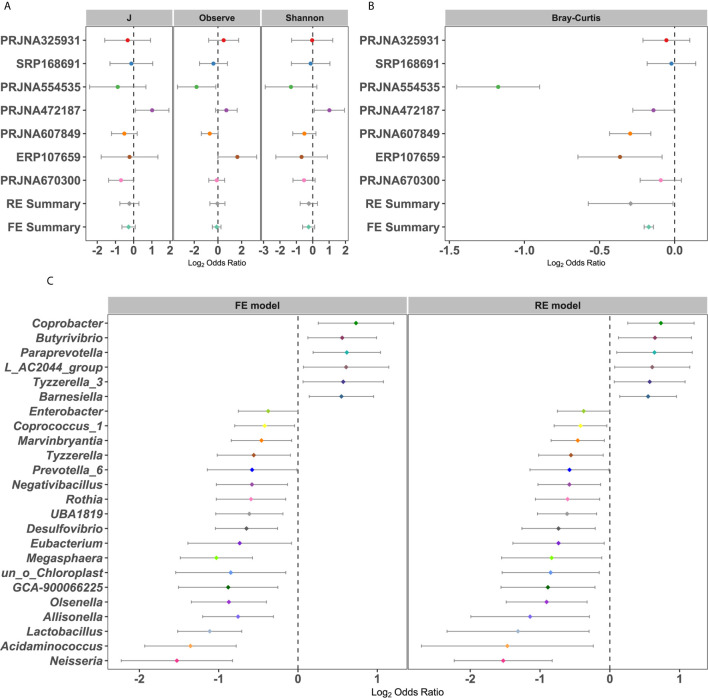
Forest plot of **(A)** the alpha diversity metrics and **(B)** Bray-Cutris distances between the individual with diabetics and the controls and **(C)** the genus metrics (Among them, the full name of *L_AC2044_group* is *Lachnospiraceae_AC2044_group*). The error bar depicts the 95% confidence interval. The value less than 1.0 (left side of the dashed line) depicts that the metric is higher in T2DM than the control. The values bigger than 1.0 (right side of the dashed lines) depicts that the metric is lower in T2DM than the control. There were significantly difference between the case and the control, if there was no cross between the dashed line and the error bar.

The differences in alpha diversity metrics between T2DM patients and controls were first analyzed. When calculating the odds ratios (ORs), none of the ORs of alpha diversity metrics were significantly higher than 1.0 for T2DM in either the RE model or FE model with low heterogeneity (**Figure 2A**), indicating nonsignificant differences in microbial alpha diversity between T2DM patients and controls. Even compared within individual studies, significantly higher microbial richness in controls than T2DM was observed in only 2 of 7 studies, while significantly higher Shannon diversity and evenness were observed in only one study (**Supplementary Table 3A**). However, when measuring differences in the entire community between T2DM and controls by PERMANOVA, significant differences in overall communities between T2DM and healthy individuals were obtained in 6 of 7 studies (**Supplementary Table 3B**). Again, by calculating the ORs based on the Bray-Curtis metric in each study, we found significant bacterial community differences between T2DM and controls in both RE models and FE models with high heterogeneity (**Figure 2B**).

To further identify the significantly different taxa between healthy controls and T2DM patients, we calculated the ORs and relative abundance of all common taxa in each study (**Figures 2C** and **S2A–S5B**). Taxonomic abundances of bacterial phyla grouped by individual study showed consistent trends: increased relative abundances of Firmicutes (class Negativicutes or order Selenomonadales or family Veillonellaceae) and Actinobacteria (class Actinobacteria) and decreased relative abundances of Bacteroidetes (class Bacteroidia or order Bacteroidales) in patients with T2DM, which coincided with the RE model in our pooled meta-analysis (**Figures S2A–S5B**). The relative abundance and OR values of other species, includingbacterial phyla, class, order, and family, were depicted in **Figures S2A–S5B**, respectively. At the genus level, a total of 24 genera were identified as significantly associated with T2DM (**Figure 2C**). Six genera had significant ORs higher than 1.0 for the absence of diabetes in the RE and FE models, including *Barnesiella*, *Butyrivibrio*, *Coprobacter*, *Tyzzerella 3*, and *Paraprevotella*. Eighteen genera possessed significant ORs lower than 1.0 for the presence of diabetes, three of which were thought to be harmful to humans, including *Desulfovibrio*, *Enterobacter*, and *Neisseria*. In addition, there were some genera, such as *Lactobacillus*, *Prevotella_6*, and *Eubacteria* (58), which were beneficial to the human. These results showed that there were dependable and significant community-wide changes in the bacterial community structures of diabetic patients.

### Metagenomic T2DM Classification Models

**Figure 3:**
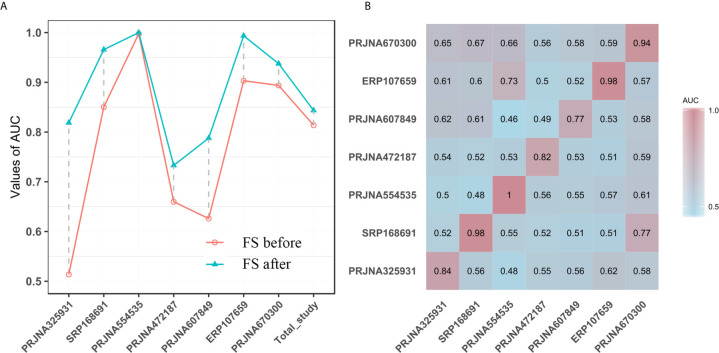
**(A)** The performances of models to classify the case and the normal before and after feature selection (FS) based on common OTUs; The horizontal ordinates represent the seven studies and the total study. The vertical coordinates depict the AUC of the individual study. **(B)** T2DM classification accuracy resulting from cross-validation within each study (the boxed along the diagonal) and study-to-study model transfer (external validations off the diagonal) as measured by the AUROC for the classification models trained on OTUs. The color of the scale bar on the right represents the AUC value.

To determine whether unique OTUs or genera could serve as biomarkers to classify patients with diabetes, we constructed two separate RF classifiers by employing a two-step procedure methodology. With the dimension decreasing from each study, the AUC value increased to varying degrees for the prediction of common OTUs and genera for seven studies (**Figures 3A** and **S6A**). Notably, these AUC values for each study were improved and reached peaks of 16% (PRJNA607849) and 27% (PRJNA325931) for OTUs and genera, respectively. The sensitivity and specificity of the total study for detection based on the cross-validation set using common OTUs were 78% (95% CI 73.8–82.1%) and 75.7% (95% CI 71.6–79.7%; AUC=0.84), respectively, for conducting feature selection, compared to 76.8% (95% CI 72.7–80.9%) and 73.6% (95% CI 70.1–77.1%; AUC=0.82), respectively, for nonconducting feature selection (**Figure 3A**). When using the common genera, the sensitivity and specificity were 77.3% (95% CI 74.0–81.0%) and 77.2% (95% CI 73.0–81.4%; AUC=0.85), respectively, for feature extraction, compared to 77.3% (95% CI 73.9–80.7%) and 74.6% (95% CI 70.4–78.8%; AUC=0.83), respectively, for no feature extraction (**Figure S6A**).

**Figure 4:**
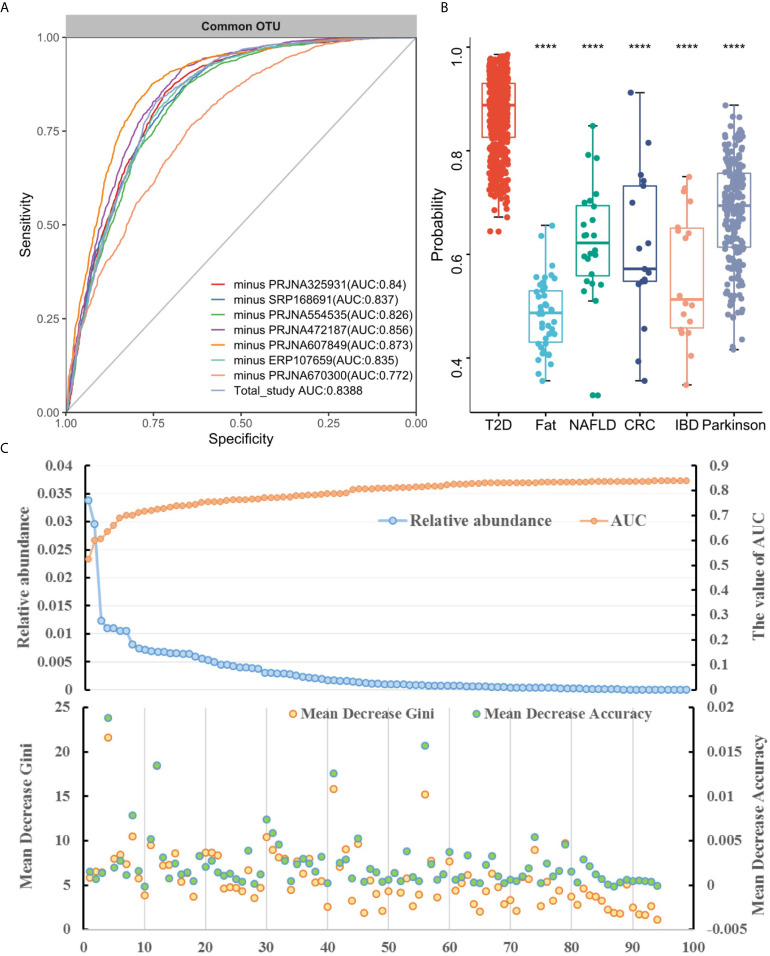
Cross-study performance of statistical models based on OTUs. **(A)** ROC of data from all other studies are combined for training (LOOS validation). **(B)** The prediction probability relative to T2DM classification models trained on fecal samples from patients with other conditions. The "****" indicates that the predictive performance of the model between other diseases and diabetes is significantly different (t-test, *p* < 0.05). **(C)** The relative abundance, AUC value and feature ranking of the selected 96 OTUs based on two-step feature selection.

Subsequently, we assessed how well the classifier trained on one study can be generalized to the other six studies. Cross-validation performance as quantified by AUC showed poor prediction performance of other studies on the predictor of one single study [median AUC = 0.58, ranging in (0.45, 0.78) for OTUs and median AUC = 0.59, ranging in (0.44, 0.77) for genera], compared to the single study’s own test set [median AUC = 0.94, ranging in (0.77, 1.0) for OTUs and median AUC = 0.95, ranging in (0.73, 1.0) for genera] (**Figures 3B** and **S6B**). We further assessed whether including data from all but one study in model training could improve prediction in the remaining hold-out study (LOOS validation). The LOOS performance of OTU-level models ranged from 0.74 to 0.85, while the LOOS performance of genus-level models ranged from 0.75 to 0.87 (**Figures 4A** and **S7**). These results suggest that the inclusion of multiple studies in the training set of a classifier can substantially improve its predictive performance relative to models trained on data from a single study. Then, by performing feature importance ranking on features obtained by feature screening based on shared OTUs of the total study (**Figure 4B**), we found that this model ranked OTUs belonging to *Dorea*, *Clostridium_sensu_stricto_1*, and *Lactobacillus* as the top 3 features in terms of mean decrease accuracy (**Table S4**). Meanwhile, we assessed the prediction performance of our T2DM classifiers based on studies for colorectal cancer (45), Parkinson’s disease (46), inflammatory bowel disease (47), NAFLD (48), and fat patients (49) (**Figure 4C**). Interestingly, we found that our OTU classification models were significantly improved over those observed for classifiers trained on other diseases, calibrated to have an average value of 0.87± 0.01 on T2DM data sets (t-test, *p*<0.05, **Figure 4C**). However, the average value of predicted probabilities on other disease data sets ranged from 0.48± 0.02 to 0.68 ±0.01. At the same time, the difference test found that diabetes is significantly different from other diseases (**Figure 4C**).

### The Correlation of Featured Genera With Probiotics

**Figure 5:**
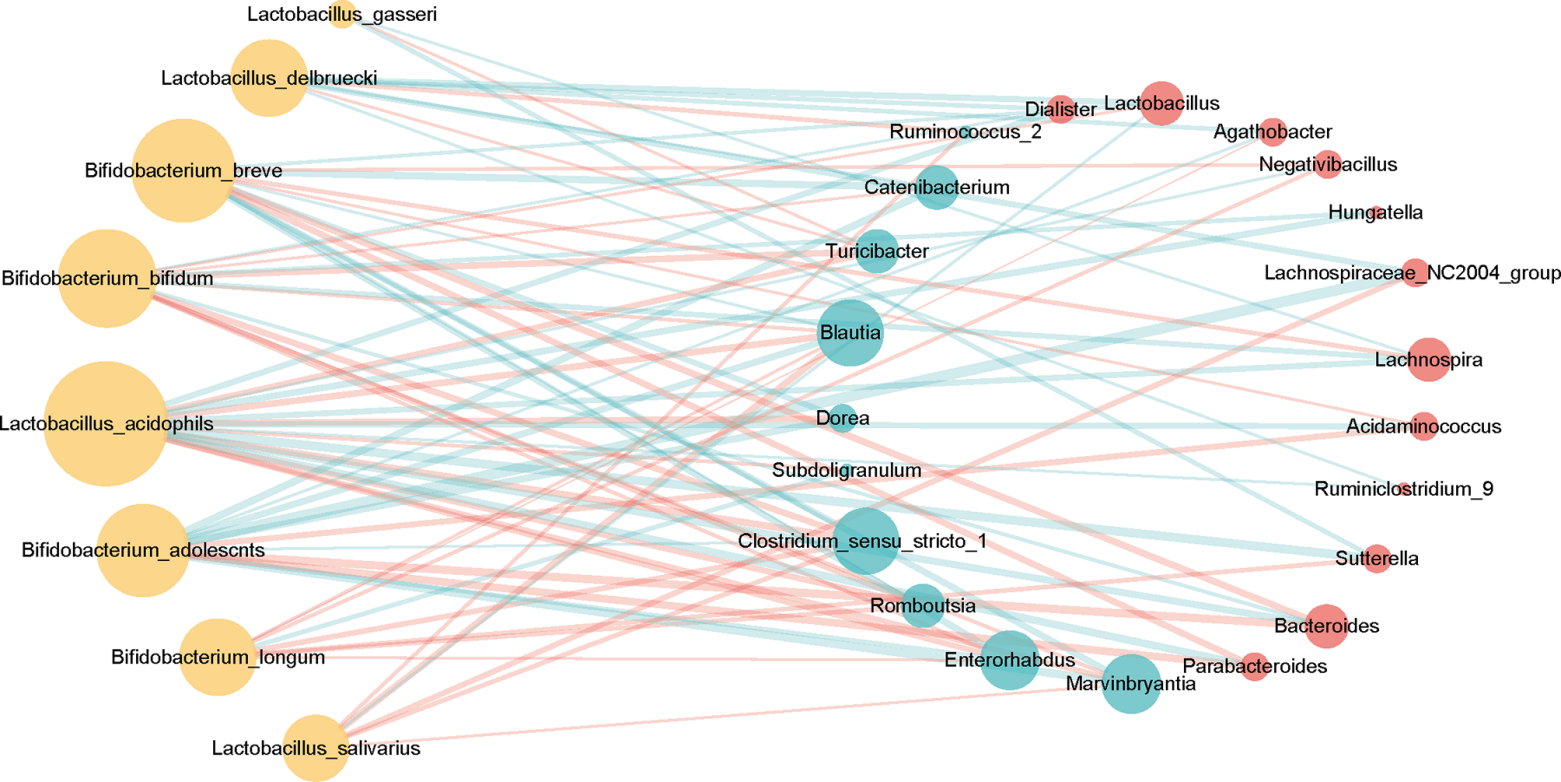
Associations between T2DM-enriched or depleted genera and probiotics. Differentially enriched 24 genera (*q*-value <0.05, FDR-controlled Wilcoxon rank-sum test, **Table 2**) were analyzed for associations with probiotics. Twenty-two of the 24 genera with significant correlation [P-value <0.05, abs(r-value)>0.1] were selected for the visualization. T2DM-enriched genera are represented by red circles, and control-enriched genera are represented by cyan circles. The size of each circle indicates connectivity. Green lines indicate positive association, and red lines—negative associations.

In the context of diabetes mellitus, experimental and clinical studies have demonstrated that different species of bacteria reduce oxidative stress, showing antidiabetic effects (59). Thus, to further discuss the effects of probiotics on metabolic control in T2DM subjects, we studied the interaction between selected genera and probiotics. Based on the 149 differentially enriched genera and the top 30 genera corresponding to the importance of OTUs selected based on feature selection, 24 overlapping genera were selected for downstream analysis (**Tables 2** and **S5**). In addition to distinguishing between individuals with and without T2DM, twenty-two of the 24 genera showed associations with a number of probiotics (Spearman correlation *p* < 0.05, abs(r-value) >0.1, **Figure 5**). For example, T2DM-depleted genera, including *Clostridium_sensu_stricto_1*, *Blautia*, and *Dorea*, were positively correlated with *Bifidobacterium breve* and *Bifidobacterium adolescnts* and were negatively correlated with *L. acidophils*. Meanwhile, *Clostridium_sensu_stricto_1*, *Blautia*, and *Lactobacillus* were correlated negatively with *Bifidobacterium bifidum*. Also, *Lactobacillus* that was enriched in T2DM patients was positively correlated with *Lactobacillus delbruecki*, *B. breve*, and *Lactobacillus salivarius*. By observing correlations with probiotics, supplementation with specific probiotics may effectively regulate the gut microbiota and improve T2DM, implying that balancing the intestinal microecology could provide a new prevention and treatment method for T2DM patients. Indeed, studies have shown that there is a significant association between certain genera and diabetes.

**Table 2:** Importance, odd ration, confidence interval, and relative abundance of the 24 genera selected for the RF model for T2DM based on all samples.

Genera	Mean decrease Gini	OR	CI_ub	CI_lb	Abundance (%)	Abundance (%)	*P*-value
					in DM	in the Normal	
*Romboutsia*	42.283	1.486	0.419	5.276	0.965 ± 2.89	2.842 ± 5.01	1.22E-38
*Dorea*	25.959	1.326	0.889	1.977	0.866 ± 1.66	1.521 ± 1.78	5.31E-27
*Lactobacillus*	21.221	0.402	0.199	0.813	1.251 ± 5.21	0.330 ± 1.23	6.32E-07
*Clostridium_sensu_stricto_1*	16.924	1.147	0.462	2.850	1.416 ± 4.61	4.270 ± 7.53	1.32E-25
*Blautia*	14.026	0.996	0.632	1.570	2.699 ± 5.45	5.963 ± 7.85	3.94E-19
*Agathobacter*	12.796	0.677	0.276	1.662	0.894 ± 1.89	0.868 ± 1.60	8.97E-06
*Subdoligranulum*	12.743	1.022	0.653	1.599	2.264 ± 3.77	3.059 ± 5.36	2E-08
*Parabacteroides*	11.993	1.076	0.349	3.312	1.942 ± 4.14	0.688 ± 1.37	1.93E-18
*Marvinbryantia*	11.899	0.727	0.558	0.947	0.104 ± 0.26	0.154 ± 0.25	1.11E-11
*Ruminiclostridium_9*	11.706	0.708	0.393	1.273	0.163 ± 0.41	0.079 ± 0.15	1.1E-06
*Ruminiclostridium_5*	11.188	1.225	0.688	2.178	0.109 ± 0.20	0.125 ± 0.18	0.001016
*Bacteroides*	11.113	0.895	0.695	1.152	19.200 ± 20.57	11.896 ± 18.69	3.48E-16
*Ruminococcus_2*	10.370	1.444	0.923	2.259	1.604 ± 4.09	2.304 ± 4.18	1.96E-07
*Sutterella*	10.159	0.680	0.449	1.030	0.809 ± 1.85	0.249 ± 0.78	1.93E-19
*Acidaminococcus*	10.157	0.361	0.154	0.848	0.456 ± 2.11	0.013 ± 0.12	2.45E-17
*Enterorhabdus*	9.869	1.006	0.763	1.325	0.189 ± 0.69	0.463 ± 1.55	9.33E-17
*Lachnospira*	9.102	1.166	0.856	1.588	0.462 ± 1.41	0.378 ± 1.06	0.007919
*Dialister*	8.702	0.651	0.336	1.258	1.717 ± 4.87	1.066 ± 4.05	0.000239
Lachnospiraceae_NC2004_group	8.347	0.879	0.500	1.543	0.414 ± 1.37	0.376 ± 1.19	6.86E-10
*Hungatella*	7.428	0.662	0.366	1.195	0.093 ± 0.45	0.018 ± 0.05	5.21E-13
*Catenibacterium*	7.403	0.852	0.512	1.418	0.889 ± 2.73	1.757 ± 4.25	6.59E-09
*Turicibacter*	7.275	1.293	0.774	2.161	0.174 ± 0.63	0.516 ± 1.34	1.58E-13
*Negativibacillus*	6.485	0.669	0.490	0.912	0.033 ± 0.13	0.015 ± 0.06	1.25E-07
*Neisseria*	5.859	0.347	0.213	0.565	0.013 ± 0.08	0.001 ± 0.005	5.61E-09

## Discussion

This study systematically evaluated the differences in intestinal flora between T2DM patients and healthy controls based on 1143 samples from China (16, 31), Colombia (32), Pakistan (28), Africa (30), Mexico (29). We observed significant differences in overall microbial communities (beta diversity) between T2DM and the controls but no differences in alpha diversity indexes. Various OTUs and bacterial genera with significant odds ratios were identified for T2DM. Through ranking abundance and stepwise feature selection, the RF models based on single studies maintained their accuracy in other studies. By training on multiple studies, we further improved the accuracy and specificity of models for T2DM. Finally, the correlations between T2DM-associated genera and probiotics provide support for diabetes intervention or prevention by probiotics.

Our sequence-based analysis portrayed non-significant differences in OTU richness, evenness and Shannon diversity index between T2DM cases and controls. This is consistent with the results of most included studies, among which only two studies reported significant differences in Chao1 in patients with T2DM compared to controls (16, 29) and one study reported significant differences in observed and Shannon indicators (30). Meanwhile, comparing alpha diversity based on OR values, the PRJNA472187 study showed that diabetic patients tended to have less evenness and Shannon diversity than controls, while other studies showed the opposite trend. The absence of consistency indicates no significant differences in alpha diversity between T2DM and healthy controls, possibly due to the relatively small sample sizes or methodological variability for generating microbiome data. Significant differences in beta diversity metrics between T2DM cases and controls were reported by five studies (16, 28–31). There were also significant differences between T2DM and HC samples in a meta-analysis with higher heterogeneity for the RE model. Meanwhile, we observed distinct clustering of 7 individual studies by Bray-Curtis metrics (*p*<0.001). This heterogeneity in 7 studies of cases and controls may be due to the methodological, clinical, or study heterogeneity (geography, ethnicity, diet) of the included studies.

Meanwhile, studies have reported a positive correlation between *Lactobacillus* and T2DM (19, 20, 44, 60, 61). In agreement with these results, *Lactobacillus* and *Eubacteria* were significantly enriched with diabetes in the meta-analysis. *Eubacteria* have been reported as SCFA producers, including propionate and butyrate. Sanna et al. found that butyrate produced by intestinal microorganisms can improve the body’s insulin response and further promote immune modulation, while propionate abnormalities can increase the risk of T2DM (22). Moreover, the reported microbial profiles of patients with T2DM were analogical across the 7 included studies. Chávez-Carbajal et al. reported that the phylum Actinobacteria was highly abundant in patients with T2DM, whereas the phylum Bacteroidetes was less abundant (29). Five studies and some literatures reported the predominance of Firmicutes in T2DM patients (15, 29, 62). In our pooled meta-analysis, we found a consistent trend toward increased relative abundances of the phyla Firmicutes (class Negativicutes or family Veillonellaceae) and Actinobacteria and decreased relative abundances of Bacteroidetes (class Bacteroidia or family Bacteroidaceae) for T2DM. If dysbiosis with increased Actinobacteria and decreased Bacteroidetes is associated with T2DM, then measures to balance these taxa by FMT or other methods may be beneficial and feasible to improve T2DM.

We developed random forest classification models using microbiota data at the OTU and genus levels. Through extensive and statistically rigorous validation, our meta-analysis firmly establishes that gut microbial signatures are highly predictive of diabetes. In particular, 16S rRNA classifiers trained on OTU and genus profiles from multiple studies maintained a median AUROC of 0.83 [ranging in (0.77, 0.87)] and 0.84 [ranging in (0.75, 0.87)], respectively, in six out of seven data sets compared to a single study [median AUC = 0.56, ranging in (0.52, 0.62)]. This may be attributed to the fact that the samples studied by a single center are not universal. Meanwhile, our RF analysis identified several OTUs related to *Dorea*, *Clostridium_sensu_stricto_1*, and *Lactobacillus* as the most important features for predicting diabetes. The relationship between T2DM-associated (enriched or depleted) genera and probiotics shows that *Clostridium_sensu_stricto_1* and *Blautia* were positively correlated with *B. breve*, and that *Lactobacillus* enriched in T2DM patients was correlated negatively with *B. bifidum*. Previously, *Clostridium_sensu_stricto_1* was reported to be negatively correlated with insulin (63), C-peptide and triacylglycerol (64). *Lactobacillus* was significantly positively correlated with glucose and glycated hemoglobin (23). *L. reuteri* (65) used as a monoprobiotic have been reported to improve T2DM-related symptoms in humans. Our results support prior work suggesting adjustment of the intestinal microecology to provide new prevention and treatment strategies for T2DM.

There were limitations in this study. First, the study methodology in the included studies varied. These studies were performed with different sequencing platforms and sequencing regions, and differences in research methods have certain effects on the intestinal microbiota. Second, the included reports had relatively small sample sizes, with three of the seven studies recruiting more than 50 participants with T2DM. Some authors were reluctant to share data. Third, the studies included in our analysis used 16S rRNA sequencing to analyze the changes in bacterial groups, which underestimated the complexity of the gut microbiota. Despite these limitations, we systematically searched all raw sequencing data and meta-data and analyzed them in a suitable and uniform manner to minimize heterogeneity, which is important to detect alterations in the gut microbiota in patients with diabetes.

In summary, our study analyzed diverse fecal 16S rRNA gene sequencing datasets in a uniform manner and revealed shifts in fecal bacterial diversity and taxa in T2DM. By selecting bacterial features and building an RF model, we raise the possibility of a fecal bacterial mode of monitoring gut health and a complementary approach for risk assessment of T2DM. Furthermore, by analyzing the interaction between T2DM-associated genera and probiotics, we provide evidence for the therapeutic potential of probiotics applied in T2DM to restore and maintain a healthy gut microbiota state.

## Data Availability Statement

The original contributions presented in the study are included in the article/**Supplementary Material**. Further inquiries can be directed to the corresponding authors.

## Author Contributions

BZ, CX, KH, and MC conceived of the study. JH, ZC, AL, LY, QZ, QC, CY, ZW, DZ, and FC were responsible for collecting published data sets, YQ, MC, JH, and BZ were involved with the meta-analysis, YQ, MC, CX, ZC, and KH completed the statistical analysis, and YQ, MC, JH, CX, KH, and BZ wrote the paper. All authors contributed to the article and approved the submitted version.

## Funding

This work was supported by the National Natural Science Foundation of China (81800517, 81900541, 81802376, and 82004433), Postdoctoral Research Foundation of China (2018M632588, 2018M632585, and 2019T120559), and National Health Commission of China NHC Key Laboratory of Health Economics and Policy Research (NHC-HEPR2019003).

## Conflict of Interest

AL was employed by company Xiamen Treatgut Biotechnology Co., Ltd.

The remaining authors declare that the research was conducted in the absence of any commercial or financial relationships that could be construed as a potential conflict of interest.

## Publisher’s Note

All claims expressed in this article are solely those of the authors and do not necessarily represent those of their affiliated organizations, or those of the publisher, the editors and the reviewers. Any product that may be evaluated in this article, or claim that may be made by its manufacturer, is not guaranteed or endorsed by the publisher.
