# Supplementary material for: Gut Bacterial Characteristics of Patients With Type 2 Diabetes Mellitus and the Application Potential
Source: Front Immunol. 2021 Aug 12;12:722206. doi: 10.3389/fimmu.2021.722206 (PMC8415158; doi:10.3389/fimmu.2021.722206)
Supplement: Supplementary file 1 [file DataSheet_1.docx]

Supplementary Materials

# Supplementary Figures and Tables

## Supplementary Figures


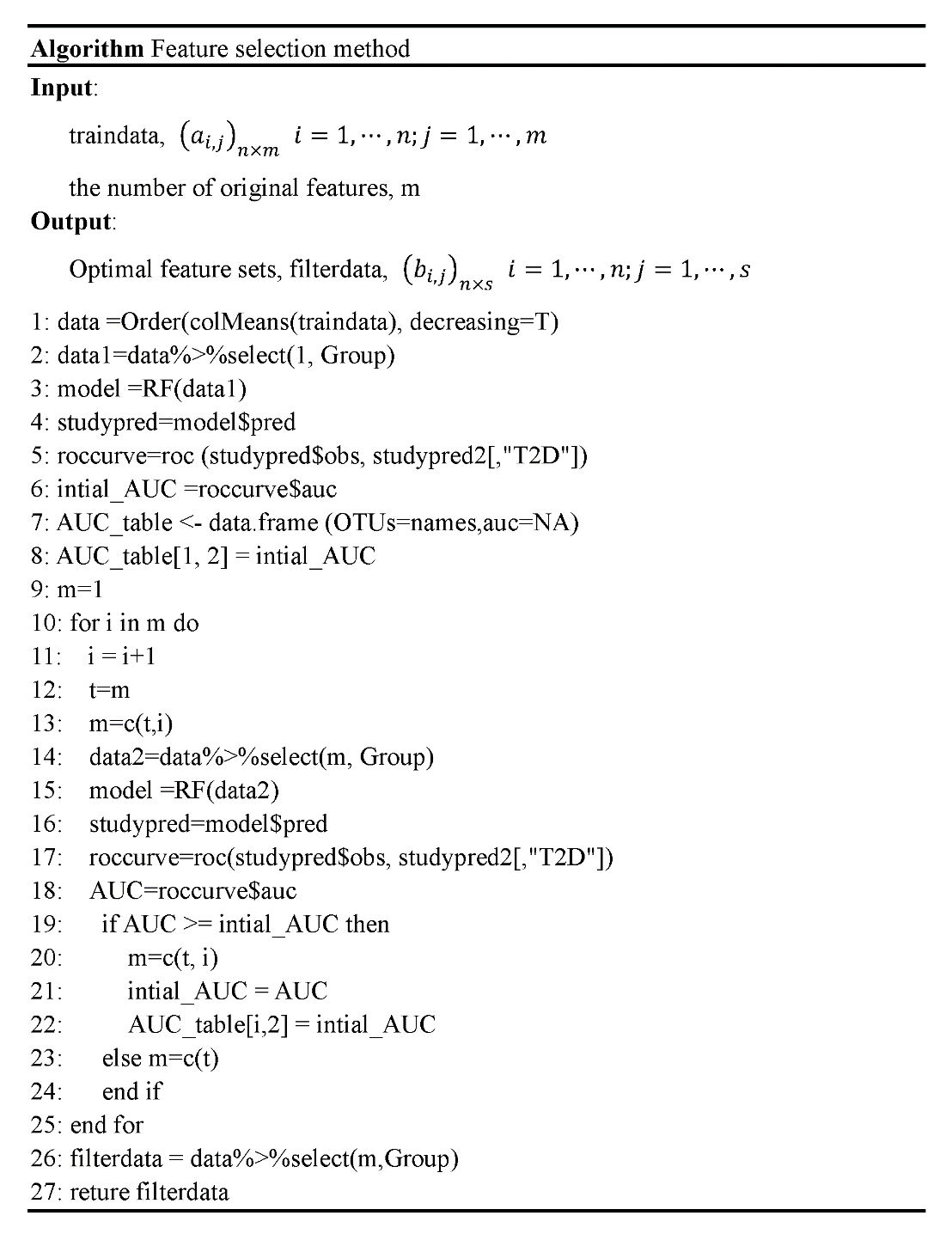


**Supplementary Figure 1.** The pseudocode of two-step feature selection algorithm.


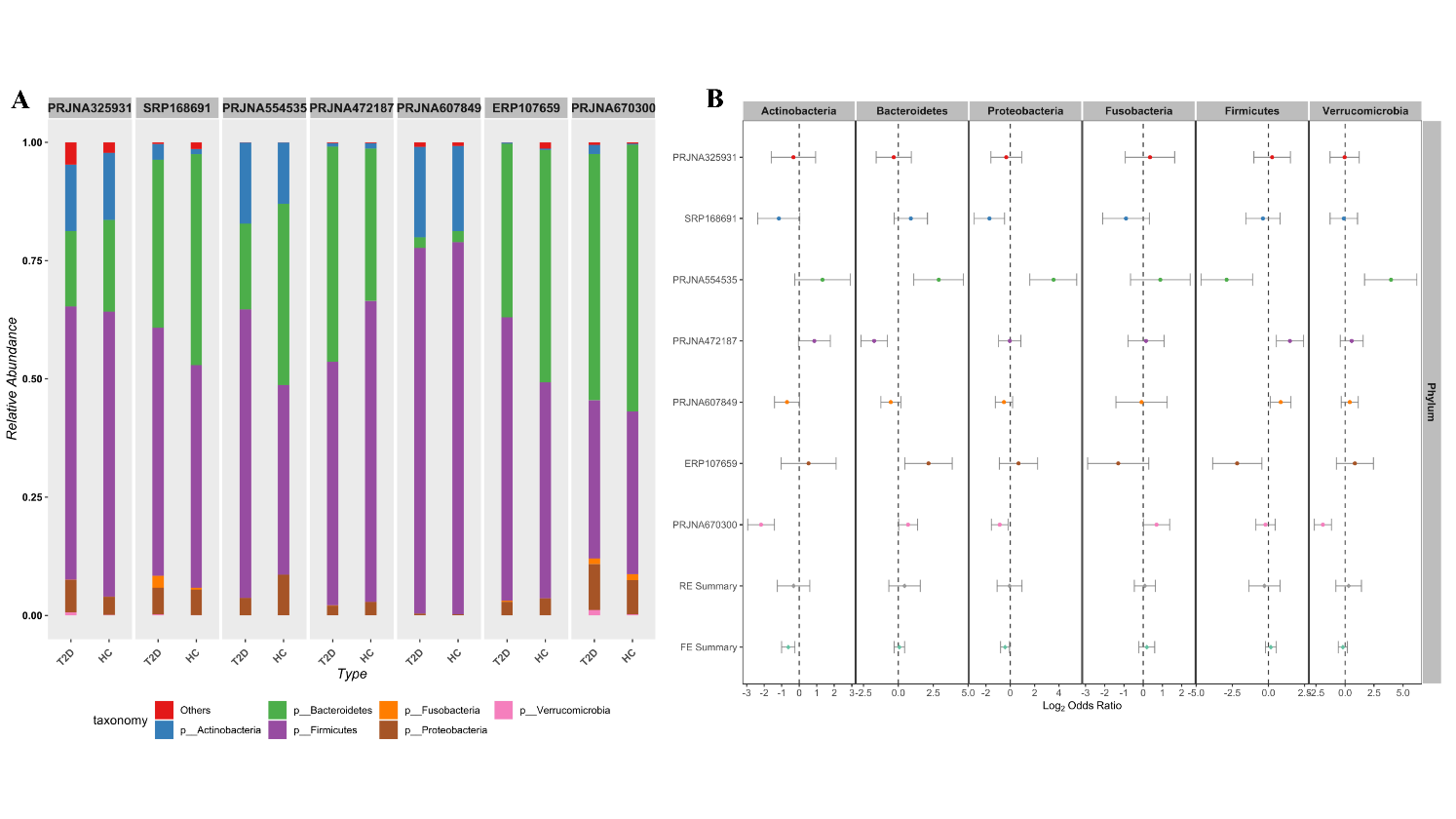


**Supplementary Figure 2.** The relative abundance (A) and OR value (B) of bacteria phyla between T2DM and controls in different research projects.


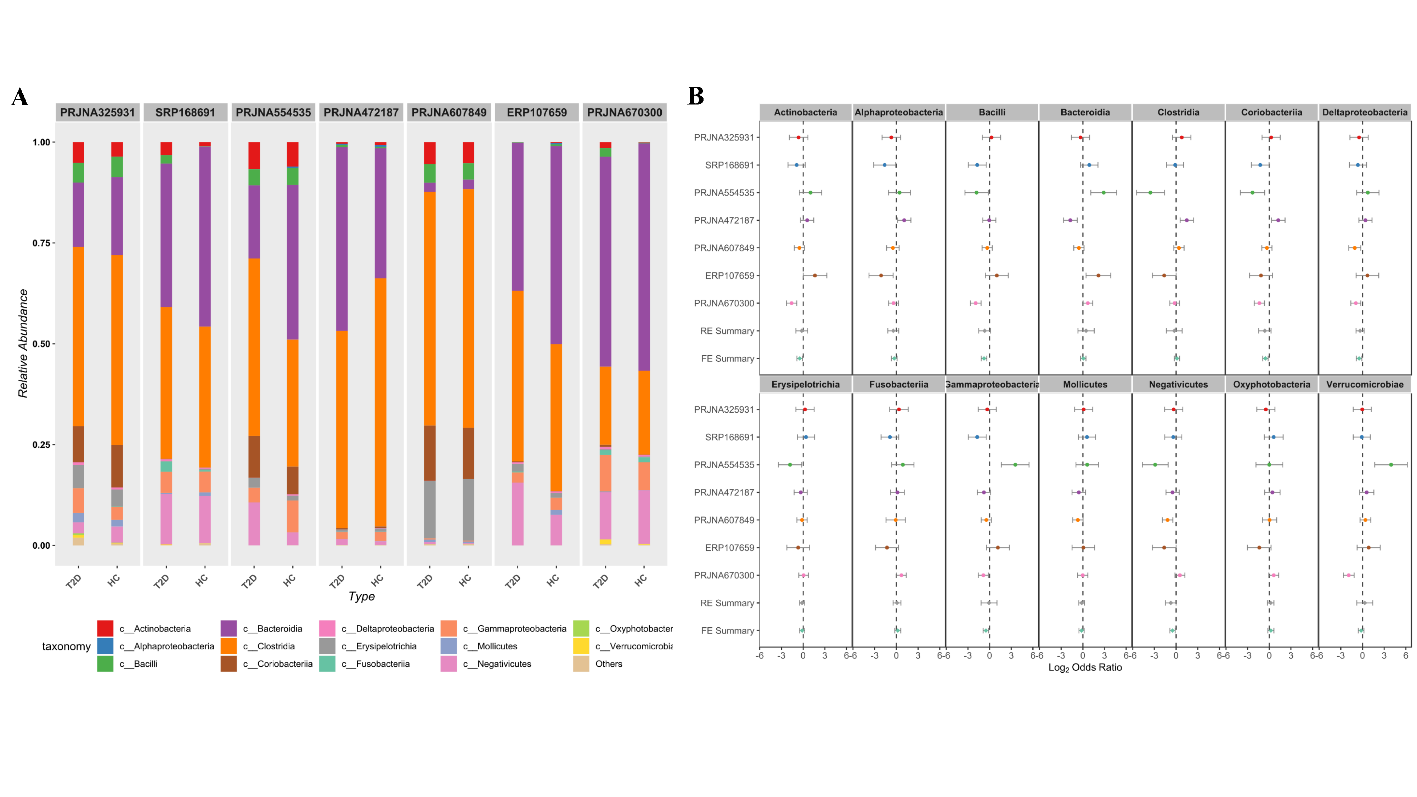


**Supplementary Figure 3.** The relative abundance (A) and OR value (B) of bacteria class between T2DM and controls in different research projects.


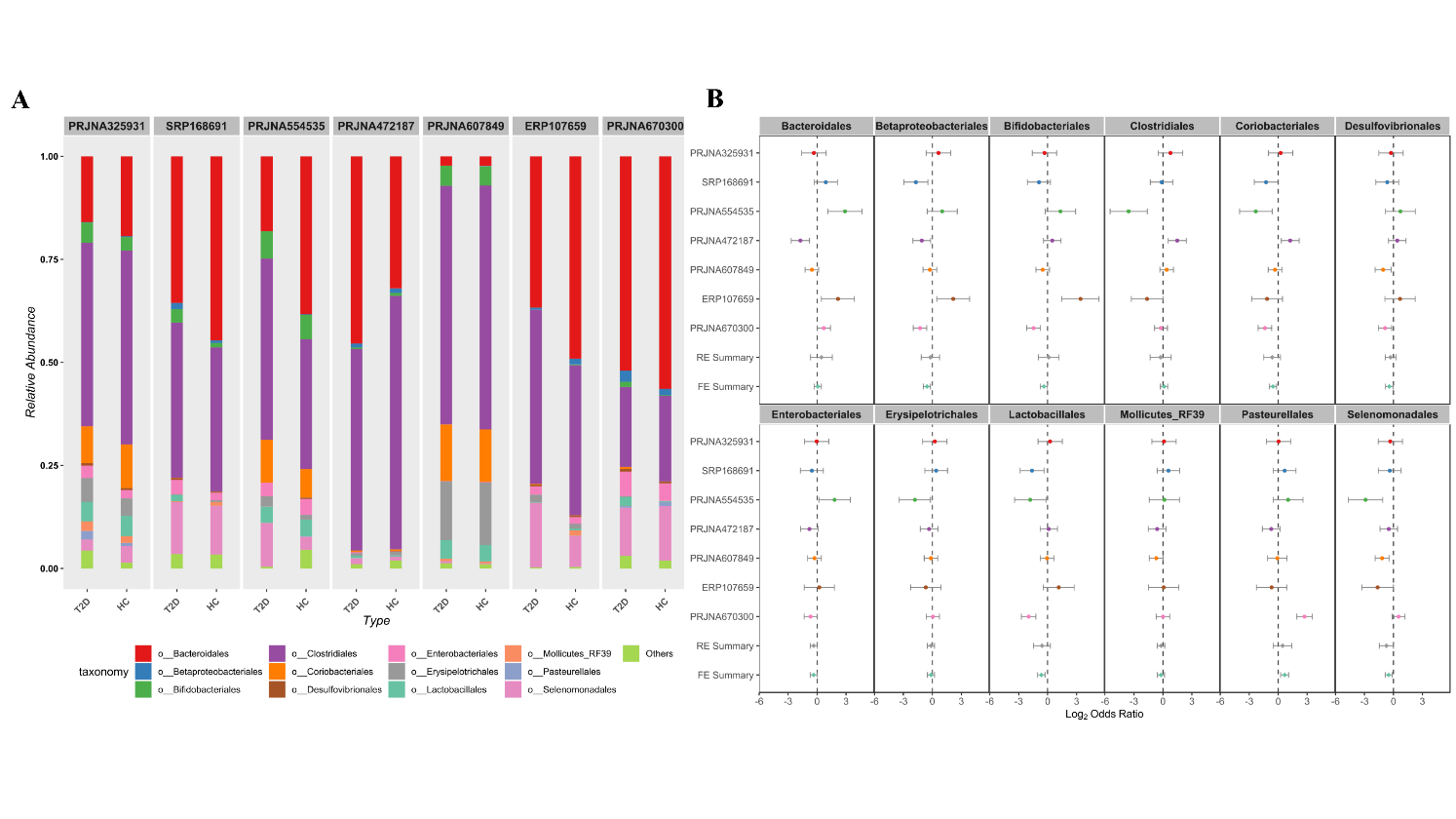


**Supplementary Figure 4.** The relative abundance (A) and OR value (B) of bacteria order between T2DM and controls in different research projects.


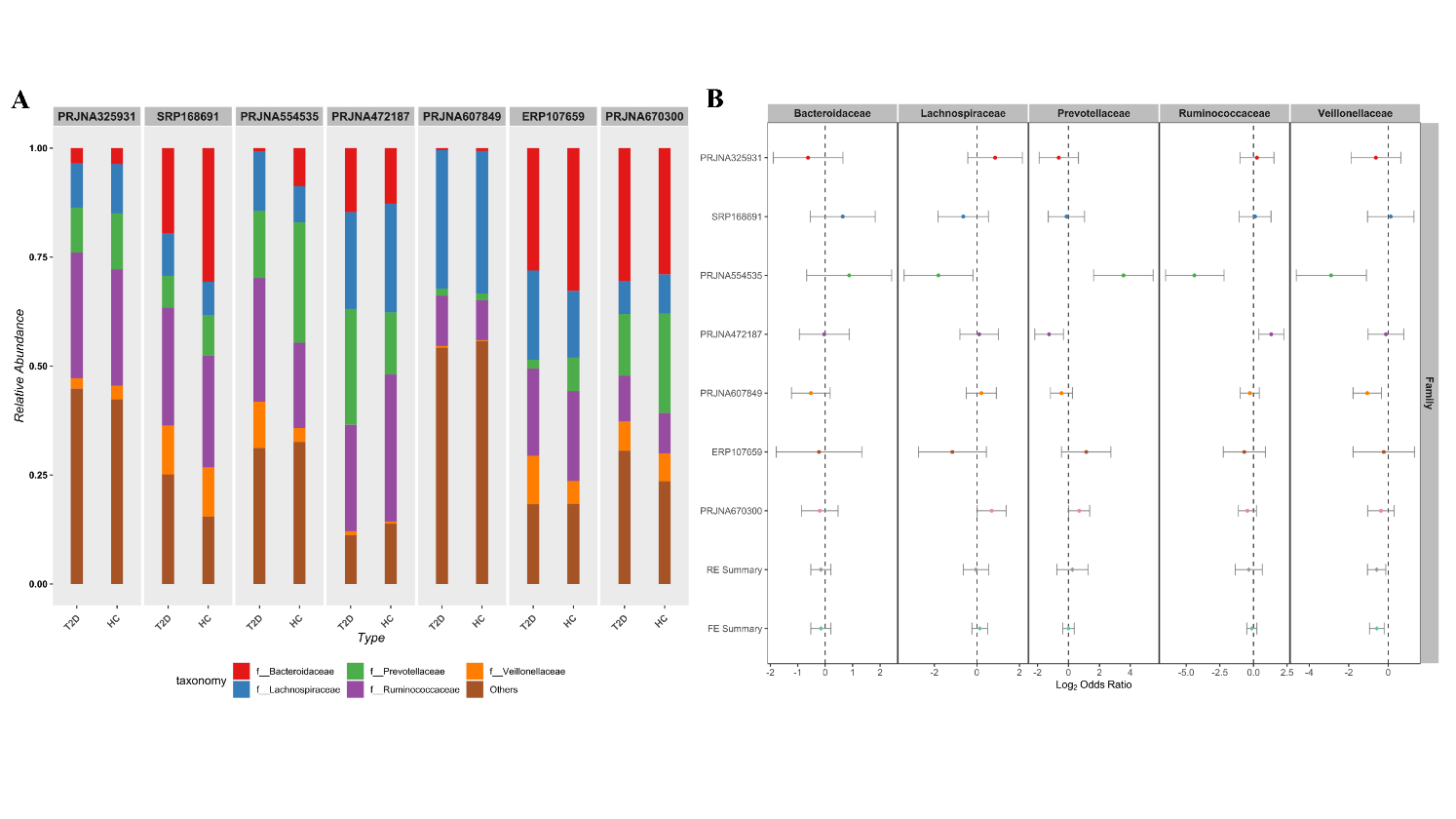


**Supplementary Figure 5.** The relative abundance (A) and OR value (B) of bacteria family between T2DM and controls in different research projects.


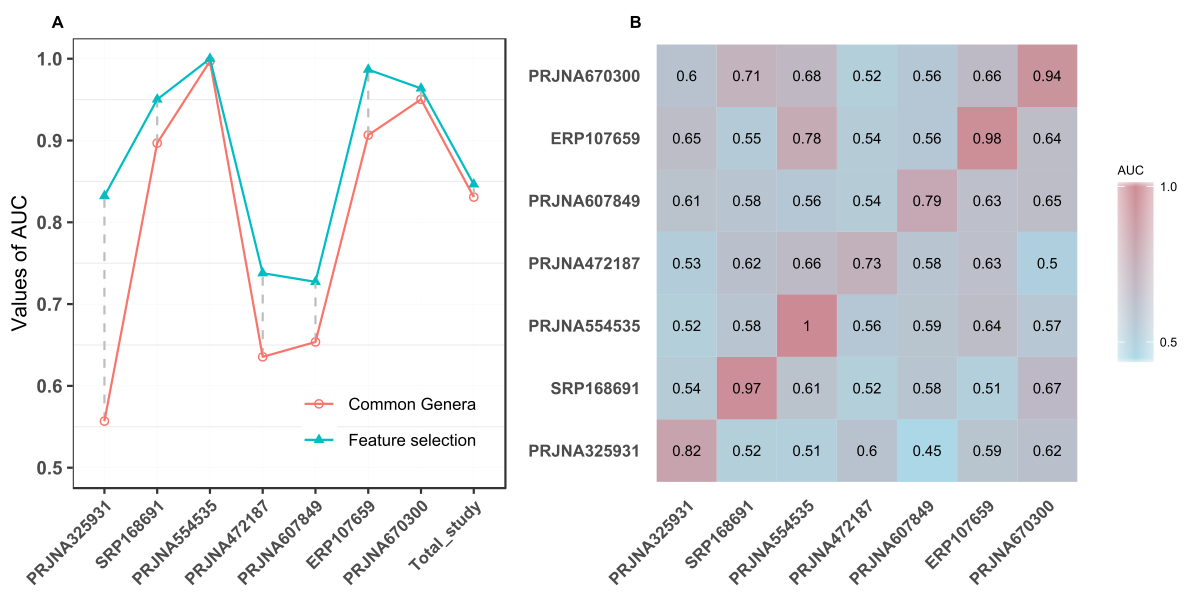


**Supplementary Figure 6.** (A) The performances of models to classify the case and the normal based on common genera; The horizontal ordinates represent the seven studies and the total study. The vertical coordinates depict the AUC of the individual study before and after feature selection. (B) T2DM classification accuracy resulting from cross-validation within each study (the boxed along the diagonal) and study-to-study model transfer (external validations off the diagonal) as measured by the AUROC for the classification models trained on genera.


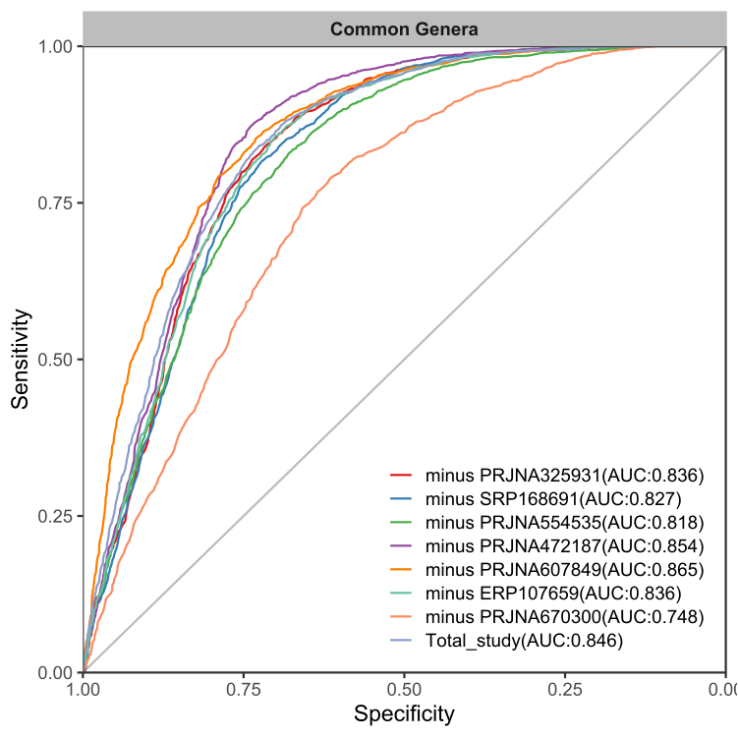


**Supplementary Figure 7.** ROC of data from all other studies are combined for training (LOOS validation) based on common genera.

## Supplementary Tables

**Supplementary Table S1**. Characteristics of the fecal 16S rDNA sequencing studies included in the meta-analysis.

| Year | Country | Source | Health | PreD | T1D | T2D | DNA extraction | Region | Seq platform |
| --- | --- | --- | --- | --- | --- | --- | --- | --- | --- |
| 2010 | Danish | NA | 18 | 0 | 0 | 18 | QIAamp DNA Stool | v4-v6 | GS FLX |
|  |  |  |  |  |  |  | Mini kit |  |  |
| 2013 | China | SRA068175 | 44 | 64 | 0 | 13 | -- | V3-V5 | 454 GS FLX |
|  |  |  |  |  |  |  |  |  |  |
| 2015 | Moscow | NA | 48 | 24 | 0 | 20 | MiSeq Reagent | V3-V4 | Miseq |
|  |  |  |  |  |  |  | Kitv2 |  |  |
| 2015 | Mexico | NA | 15 | 20 | 0 | 14 | QiaAMP mini | V4 | Miseq |
|  |  |  |  |  |  |  | stool kit |  |  |
| 2016 | Colombia | PRJNA325931 | 84 | 0 | 0 | 28 | QIAamp DNA | V4 | Miseq |
|  |  |  |  |  |  |  | Stool Mini Kit |  |  |
| 2016 | Italian | WebSite | 13 | 0 | 0 | 40 | DNeasy Blood & | v3-v4 | MiSeq |
|  |  |  |  |  |  |  | Tissue Kit (Qiagen) |  |  |
| 2017 | China | NA | 20 | 0 | 0 | 20 | QIAamp DNA | V6 | Hiseq |
|  |  |  |  |  |  |  | Stool Mini Kit |  |  |
| 2017 | Japanese | NA | 12 | 0 | 0 | 10 | -- | v3-v4 | MiSeq |
|  |  |  |  |  |  |  |  |  |  |
| 2018 | China Taiwan | NA | 50 | 0 | 0 | 50 | QIAamp Fast DNA | -- | qPCR |
|  |  |  |  |  |  |  | Stool Mini kit |  |  |
| 2018 | Poland | NA | 23 | 0 | 22 | 23 | Genomic Mini | -- | Miseq |
|  |  |  |  |  |  |  | AX Stool Spin |  |  |
| 2018 | Spain | NA | 13 | 0 | 15 | 15 | QIAamp DNA Stool | V2–V3 | 454 GS FLX |
|  |  |  |  |  |  |  | NucleoSpin Soil kit |  |  |
| 2018 | Danish | NA | 0 | 134 | 0 | 134 |  | V4 | Miseq |
|  |  |  |  |  |  |  | FastDNA® Spin Kit |  |  |
| 2019 | China | SRP168691 | 35 | 0 | 0 | 65 |  | V3-V4 | Ion S5TM |
|  |  |  |  |  |  |  | TIAGEN DNA |  |  |
| 2019 | Pakistan | PRJNA554535 | 20 | 0 | 0 | 40 | Stool kit | V3–V4 | Miseq |
|  |  |  |  |  |  |  | MagNA Pure LC |  |  |
| 2019 | Austria | PRJNA510713 | 0 | 0 | 0 | 26 | DNA Isolation Kit III | -- | Miseq |
|  |  |  |  |  |  |  | QIAamp DNA Stool |  |  |
| 2019 | German | NA | 1127 | 0 | 0 | 153 | Mini Kit | V3 | Miseq |
|  |  |  |  |  |  |  | MoBioPowerMag® |  |  |
| 2020 | Africa | PRJNA607849 | 193 | 0 | 0 | 98 | Microbiome kit | V4 | Miseq |
|  |  |  |  |  |  |  | QIAamp DNA Stool |  |  |
| 2020 | China | ERP107659 | 40 | 0 | 0 | 20 | Mini kit | V4-V5 | Hiseq |
|  |  |  |  |  |  |  | Nucleospin Microbial |  |  |
| 2020 | Japanese | DRA009950 | 97 | 0 | 0 | 97 | DNA kit | V3–V4 | Miseq |
|  |  |  |  |  |  |  | QIAamp DNA Stool |  |  |
| 2020 | China | NA | 37 | 0 | 0 | 134 | PowerSoil® DNA | V3-V4 | Hiseq |
|  |  |  |  |  |  |  | Isolation Kit |  |  |
| 2020 | China | PRJNA670300 | 95 |  |  | 281 | QIAamp DNA | V4 | Miniseq |
|  |  |  |  |  |  |  | Stool Mini kit |  |  |
| 2020 | Mexican | PRJNA472187 | 76 | 54 | 0 | 14 | PowerSoil DNA | V3 | PGM |
|  |  |  |  |  |  |  | Isolation Kit |  |  |

**Supplementary Table S2**. The BMI, sex, age and Clinical parameters of the included 7 studies.

| Study | Group | Age | Sex(F/M) | BMI | FBG(mg/dL) | TC(mg/dL) | HbA1c(mmol/moL) | HDL(mg/dL) | LDL(mg/dL) |
| --- | --- | --- | --- | --- | --- | --- | --- | --- | --- |
| PRJNA325931 | T2DM | 50±10 | 0.36 | 31.88±4.63 | 127±47 | 178±51 | 6.9±1.4 | 40±11 | 105±35 |
|  |  | /44±9 | /0.50 | /32.15±6.36 | /145±76 | /208±44 | 7.1±1.7 | /38±6 | /128±39 |
|  | HC | 47±9 | 0.43 | 31.11±4.53 | 90±10 | 187±30 | 5.6±0.3 | 44±12 | 115±28 |
| SRP168691 | T2DM | 52[43,58] | 0.86 | 25.20±3.84 | 171.9[120.96,215.28] | 91.44[77.94,103.68] | 9.42±2.49 | 22.32[18.54.26.82] | 48.6±16.2 |
|  | HC | 44[42,48] | 0.94 | 22.01±1.32 | 95.94[73.08,92.7] | 86.76[78.3,90.54] | 4.38±0.78 | 25.56[20.34,28.8] | 41.94±10.26 |
| PRJNA554535 | T2DM | 38.1±12.1 | - | 32.4±3.6 | 221.5±61.5 | 202.2±25.2 | - | 46.2±10.4 | 131.2±33.4 |
|  | HC | 37.7±12.1 | - | 22.08±3.1 | 84.8±30.4 | 124.3±31.6 | - | 36.9±8.5 | 49.2±27.7 |
| PRJNA472187 | T2DM | 50.2±5.0 | 0.5 | 28.4±4.1 | 97.2±11.0 | - | 5.9±0.2 | 47.6±9.4 | 127.4±51.4 |
|  |  | /48.1±4.7 |  | /30.8±4.6 | /199.3±108.4 |  | 8.4±2.2 | /46.9±8.6 | /137.9±37.5 |
|  | HC | 48.0±5.4 | 0.52 | 27.7±3.7 | 92.9±10.1 | - | 5.4±0.2 | 47.5±10.2 | 114.8±26.2 |
| PRJNA607849 | T2DM | 59.7±10.4 | - | 32.21±5.94 | 118[96,157] | 201.16±64.12 | 7.4[6.20,8.80] | 31[20,44] | 122.72±49.77 |
|  | HC | 54.3±13.3 | - | 30.33±6.09 | 82[76,89] | 193.20±51.34 | 5.40[5.2,5.7] | 24[13,37] | 123.10±44.08 |
| ERP107659 | T2DM | - | - | 26.57±1.99 | 130.86±25.02 | 95.04±17.82 | - | 23.04±3.96 | 48.6±8.46 |
|  | HC | - | - | 21.01±1.51 | 94.14±18.72 | 79.2±14.4 | - | 25.56±3.78 | 45.72±8.82 |

FBG, fasting blood glucose; TC, total cholesterol; HbA1c, glycated hemoglobin; HDL, high-density lipoprotein cholesterol; LDL, low-density lipoprotein cholesterol.

**Supplementary Table S3a**. The Wilcoxon test results of the alpha diversity metrics.

| diabetic_median | Normal_median | pvalue | measures | study |
| --- | --- | --- | --- | --- |
| 568 | 544 | 0.124 | Observe | PRJNA607849 |
| 4.097 | 3.972 | 0.034 | Shannon | PRJNA607849 |
| 0.641 | 0.632 | 0.034 | J | PRJNA607849 |
| 417 | 437 | 0.479 | Observe | PRJNA325931 |
| 2.552 | 2.575 | 0.945 | Shannon | PRJNA325931 |
| 0.429 | 0.422 | 0.864 | J | PRJNA325931 |
| 946.5 | 718.5 | 0.035 | Observe | PRJNA554535 |
| 4.553 | 4.174 | 0.078 | Shannon | PRJNA554535 |
| 0.661 | 0.632 | 0.226 | J | PRJNA554535 |
| 590 | 649 | 0.035 | Observe | PRJNA472187 |
| 3.944 | 4.27 | 0.072 | Shannon | PRJNA472187 |
| 0.622 | 0.654 | 0.091 | J | PRJNA472187 |
| 598 | 712 | 0.114 | Observe | ERP107659 |
| 4.051 | 4.006 | 1 | Shannon | ERP107659 |
| 0.63 | 0.621 | 0.681 | J | ERP107659 |
| 635 | 606 | 0.437 | Observe | SRP168691 |
| 3.864 | 3.747 | 0.773 | Shannon | SRP168691 |
| 0.608 | 0.596 | 0.971 | J | SRP168691 |
| 422 | 419 | 0.768 | Observe | PRJNA670300 |
| 3.279 | 3.172 | 0.127 | Shannon | PRJNA670300 |
| 0.547 | 0.527 | 0.055 | J | PRJNA670300 |

**Supplementary** **Table S3b**. The PERMANOVA results of the Bray-Curtis distances.

| Study | Fstat | R2 | Pvalue | Group |
| --- | --- | --- | --- | --- |
| PRJNA607849 | 2.22 | 0.008 | 0.0015 | diabetic |
| PRJNA325931 | 0.875 | 0.008 | 0.6671 | diabetic |
| PRJNA554535 | 4.829 | 0.077 | 0.0001 | diabetic |
| PRJNA472187 | 2.214 | 0.014 | 0.0015 | diabetic |
| ERP107659 | 1.925 | 0.033 | 0.0007 | diabetic |
| SRP168691 | 1.623 | 0.016 | 0.01 | diabetic |
| PRJNA670300 | 5.305 | 0.014 | 0.0001 | diabetic |

**Supplementary** **Table S4**. The results of the feature ranking of OTU and genera selected.

| OTUs | MDA | Genera | MDA |
| --- | --- | --- | --- |
| AB506357.1.1508:f__Clostridiaceae_1;g__Clostridium_sensu_stricto_1 | 2.47E-02 | *Romboutsia* | 3.65E-02 |
| AB559693.1.1568:f__Lactobacillaceae;g__Lactobacillus | 2.32E-02 | *Dorea* | 2.11E-02 |
| AB506177.1.1524:f__Lachnospiraceae;g__Dorea | 2.31E-02 | *Lactobacillus* | 1.25E-02 |
| KJ808500.1.1480:f__Clostridiaceae_1;g__Clostridium_sensu_stricto_1 | 1.32E-02 | *Ruminococcaceae_UCG_013* | 1.13E-02 |
| KP151214.1.1490:f__Peptostreptococcaceae;g__Romboutsia | 1.02E-02 | *Clostridium_sensu_stricto_1* | 1.06E-02 |
| DQ456246.1.1451:f__Bacteroidaceae;g__Bacteroides | 7.69E-03 | *Enterorhabdus* | 1.03E-02 |
| AGZO01000001.452.1966:f__Tannerellaceae;g__Parabacteroides | 6.52E-03 | *Blautia* | 7.29E-03 |
| ADKO01000087.185.1693:f__Bacteroidaceae;g__Bacteroides | 5.67E-03 | *Parabacteroides* | 7.13E-03 |
| FMER01000018.41984.43499:f__Ruminococcaceae;g__Ruminococcaceae_UCG-013 | 5.61E-03 | *Acidaminococcus* | 6.24E-03 |
| CDYJ01035375.5063.6592:f__Ruminococcaceae;g__Ruminiclostridium_5 | 5.01E-03 | *Bacteroides* | 5.66E-03 |
| CDZS01010489.3672.5182:f__Lachnospiraceae;g__Lachnospiraceae_NK4A136_group | 4.34E-03 | *Ruminiclostridium_9* | 5.61E-03 |
| CDZB01007134.101.1647:f__Veillonellaceae;g__Dialister | 4.08E-03 | *Eubacterium_hallii_group* | 5.33E-03 |
| AAQL01011552.755.2265:f__Lachnospiraceae;g__[Eubacterium]_hallii_group | 3.89E-03 | *Agathobacter* | 5.29E-03 |
| CP013476.575138.576663:f__Erysipelotrichaceae;g__Turicibacter | 3.89E-03 | *Sutterella* | 5.18E-03 |
| EF511181.1.1499:f__Pasteurellaceae;g__Haemophilus | 3.64E-03 | *Subdoligranulum* | 5.16E-03 |
| EF404388.1.1474:f__Ruminococcaceae;g__Faecalibacterium | 3.60E-03 | *Marvinbryantia* | 4.54E-03 |
| EU358695.1.1474:f__Peptostreptococcaceae;g__un_f_Peptostreptococcaceae | 3.52E-03 | *Catenibacterium* | 4.40E-03 |
| FMDW01000023.29634.31144:f__Lachnospiraceae;g__un_f_Lachnospiraceae | 3.46E-03 | *Hungatella* | 4.14E-03 |
| AAXA02000014.354465.355983:f__Lachnospiraceae;g__Dorea | 3.10E-03 | *Ruminococcaceae_UCG_004* | 3.67E-03 |
| ACIB02000015.660160.661676:f__Bacteroidaceae;g__Bacteroides | 2.87E-03 | *Haemophilus* | 3.65E-03 |
| EF403840.1.1478:f__Ruminococcaceae;g__Subdoligranulum | 2.83E-03 | *Ruminiclostridium_5* | 3.47E-03 |
| AM405723.1.1462:f__Eggerthellaceae;g__un_f_Eggerthellaceae | 2.82E-03 | *Dialister* | 3.46E-03 |
| GQ896710.1.1484:f__Ruminococcaceae;g__Ruminococcaceae_UCG-002 | 2.70E-03 | *Lachnospiraceae_NC2004_group* | 3.43E-03 |
| CDYL01028374.638.2136:f__Lachnospiraceae;g__Lachnospiraceae_NK4A136_group | 2.62E-03 | *Ruminococcus_2* | 3.29E-03 |
| FPLO01008166.89.1602:f__Bifidobacteriaceae;g__Bifidobacterium | 2.58E-03 | *Turicibacter* | 3.11E-03 |
| FPLO01004263.15.1513:f__Lachnospiraceae;g__Coprococcus_1 | 2.51E-03 | *Lachnospira* | 3.06E-03 |
| DQ905741.1.1519:f__Ruminococcaceae;g__Faecalibacterium | 2.48E-03 | *Neisseria* | 3.02E-03 |
| AGXY01000011.2332.3846:f__Bacteroidaceae;g__Bacteroides | 2.24E-03 | *Lachnospiraceae_ND3007_group* | 3.01E-03 |
| CZAE01000003.99809.101321:f__Bacteroidaceae;g__Bacteroides | 2.10E-03 | *Ruminococcus_torques_group* | 2.94E-03 |
| EF404796.1.1476:f__Lachnospiraceae;g__Lachnospiraceae_ND3007_group | 1.90E-03 | *Negativibacillus* | 2.65E-03 |

MDA, Mean Derease Accuracy.
